# Ataxia telangiectasia and Rad3-related inhibitors and cancer therapy: where we stand

**DOI:** 10.1186/s13045-019-0733-6

**Published:** 2019-04-24

**Authors:** Lin Mei, Junran Zhang, Kai He, Jingsong Zhang

**Affiliations:** 1 Hematology, Oncology and Palliative Care, Massey Cancer Center, Virginia Commonwealth University, 1250 East Marshall Street, Richmond, VA 23298 USA; 2 Department of Radiation Oncology, The Ohio State University, James Cancer Hospital and Solove Research Institute, 460 west 10th Avenue, Columbus, OH 43210 USA; 3 The James Thoracic Oncology Center, The Ohio State University Comprehensive Cancer Center, 494 Biomedical Research Tower, Columbus, OH 43210 USA; 4 Department of Genitourinary Oncology, H Lee Moffitt Cancer Center, 12902 Magnolia Drive, Tampa, FL 33612 USA

**Keywords:** ATR, Replication stress, DNA damage, ATR inhibitors, Cancer

## Abstract

**Background:**

The ataxia telangiectasia and Rad3-related (ATR) checkpoint kinase 1 (CHK1) pathway plays an essential role in suppressing replication stress from DNA damage and oncogene activation.

**Main body:**

Preclinical studies have shown that cancer cells with defective DNA repair mechanisms or cell cycle checkpoints may be particularly sensitive to ATR inhibitors. Preclinical and clinical data from early-phase trials on three ATR inhibitors (M6620, AZD6738, and BAY1895344), either as monotherapy or in combination, were reviewed.

**Conclusion:**

Data from ATR inhibitor-based combinational trials might lead to future expansion of this therapy to homologous recombination repair pathway-proficient cancers and potentially serve as a rescue therapy for patients who have progressed through poly ADP-ribose polymerase inhibitors.

## Background

DNA damage response (DDR) is a complex interconnected signaling network that is essential to defend human genome integrity against a variety of exogenous and endogenous genotoxic insults, such as ultraviolet radiation, ionizing radiation, or reactive oxygen species. Ataxia telangiectasia-mutated (ATM) checkpoint kinase 2 (CHK2) and ataxia telangiectasia and Rad3-related (ATR) checkpoint kinase 1 (CHK1) signals are two key pathways to initiate DDR. In response to DNA double-strand (dsDNA) breaks, the MRE11/NBS1/RAD5 complex activates the ATM-CHK2 kinase, which stabilizes p53 through phosphorylation and arrests the cell cycle at the G1/S phase checkpoint [1, 2]. When single-strand DNA (ssDNA) is produced at sites of DNA damage or stressed replication forks, replication protein A-coated ssDNA mobilizes ATR and its binding partner, ATR interacting protein (ATRIP) [3, 4]. CHK1 is subsequently phosphorylated by ATR on Ser-317 and Ser-345 [5]. Inhibitory phosphorylation by CHK1 of the phosphatase CDC25A and its subsequent proteasomal degradation leads to a decrease in CDK2 activity during the S phase [6], triggering the intra-S phase and G2/M phase checkpoints [7–9]. Given that an extended ssDNA of a stalled replication fork is a common feature of replication stress, ATR also plays a key role in replication stress response. After ssDNA is coated by RPA, ATR is recruited along with its obligatory partner, ATRIP, to initiate replication stress response. ATR-ATRIP complex activation requires TOPBP1, the trimeric RAD9-RAD1-HUS1 (9-1-1) complex, and Ewing’s tumor-associated antigen 1 (ETAA1). ATR, along with protein adaptors such as Claspin or 9-1-1 complex and interacting nuclear orphan (RHINO), subsequently phosphorylates a multitude of targets, including CHK1 [3, 17]. The downstream targets of the activated ATR-CHK1 axis are essential in suppressing replication stress [18]. ATM knock out mice have a similar ataxia telangiectasia phenotype with a high incidence of lymphoma [19], whereas homozygous elimination of ATR leads to chromosome breaks, proliferative failure in culture, and early embryonic lethality [20].

In vitro and in vivo studies demonstrated cross talks between the ATM and ATR pathways [10–12]. ATM-mediated dsDNA break processing results in regions of RPA-coated ssDNA that are then recognized by ATR. ATR is subsequently activated in response to dsDNA breaks in an ATM-dependent manner [21–23]. UV and hydroxyurea, potent activators of ATR, were also shown to phosphorylate and activate ATM in an ATR-dependent manner [24]. Depletion of ATR with doxycycline-inducible lentiviral system in ATM-deficient cells caused severe G2/M checkpoint attenuation and synthetic lethality following ionizing radiation [16]. Inhibition of ATR with small molecule inhibitor selectively sensitized ATM or p53-deficient cancer cells to cisplatin [13–15].

**Fig. 1:**
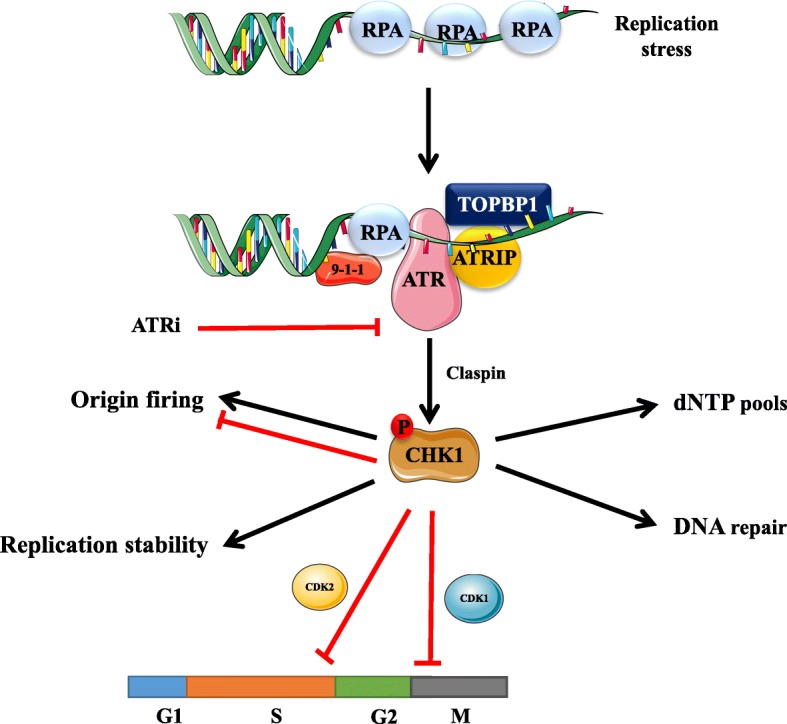
Replication stress induced ATR-CHK1 activation. ATR is activated by replication protein A (RPA)-coated single-stranded DNA (ssDNA) that arises at stalled replication fork or resected DNA double-strand break (DSB), particularly at ssDNA and double-stranded DNA (dsDNA) juncture. The recruitment of ATR-interacting protein (ATRIP) leads to recognition of ATR and RPA-ssDNA complex. Subsequently, it incorporates Rad9-Rad1-hus1 (9-1-1) and DNA topoisomerase 2-binding protein 1 (TOPBP1), leading to ATR activation. Mediated by adaptor protein claspin, ATR phosphorylates checkpoint kinase 1 (CHK1). The activation of CHK1 can prevent genomic instability. The mechanisms are either promoting or inhibiting the initiation of DNA replication (origin firing), ensuring sufficient supply of deoxynucleotides (dNTPs) pool, stabilizing replication fork and DNA repair. Its downstream molecules, cyclin-dependent kinase (CDK) 1 and CDK2, suppresses G2-M transition and slows down S phase

Several features of cancer cells may sensitize them to inhibitors that target ATR-CHK1. First, ATR-CHK1-mediated signaling is often particularly evident in cells with a defective G1 checkpoint that was caused by a mutation in p53 or a loss of retinoblastoma protein. Mutations in p53 have been reported as potential resistance mechanisms to cytotoxic chemotherapy or targeted therapies such as poly (ADP-ribose) polymerase (PARP) inhibitors [25]. In addition, premature entering into mitosis phase after abrogation of G1 and/or G2 checkpoints will lead to mitotic catastrophe, which is characterized by the formation of nuclear envelopes around individual clusters of missegregated chromosomes [26]. Blocking ATR activity with small molecular inhibitors either alone or combined with DNA-damaging agents can lead to mitotic catastrophe of cancer cells and p53-independent cell death [13, 27]. ATR and/or CHK1 suppress replication stress that arises from causes such as DNA damage and oncogene activation. Combining ATR inhibitors with DNA-damaging radiation or chemotherapy could lead to synthetic lethality, particularly in cancer cells that harbor overexpression of oncogenes like Myc [28]. Currently, there are three different ATR inhibitors under early-phase clinical development (Fig. 1). These are M6620, AZD6738, and BAY1895344. In this mini-review, we discuss the emerging clinical data that have been reported through the development of these ATR inhibitors as either mono- or combinational therapies (Table 1).

**Table 1:** Summary of ATR inhibitor-based clinical trials

ATRi	Target cancer type	Treatment	Phase	Biomarker selection	Efficacy and toxicity	Identifier
M6620 (formerly VX-970, IV)	Advanced solid tumor	Alone or with carboplatin/paclitaxel	I		Gr 3/4: 5–21% 1 pt had PR	NCT03309150
	Advanced solid tumor	Gemcitabine, cisplatin, etoposide, or carboplatin	I	*TP53* mutation of *ATM* loss	Gem, Gr 3/4, 50%; PFS, 8.0–29.3 weeks Cis, Gr 3/4, 46%; PFS, 4.1 months	NCT02157792
	Advanced solid tumor	Irinotecan	I			NCT02595931
	Small-cell cancers	Topotecan	I/II	DDR pathway mutations	Gr 3/4, 10–19%; PR + SD, 42.8%	NCT02487095
	Urothelial carcinoma	Cisplatin or gemcitabine	I/II	p53, p21, and *ERCC2* mutations		NCT02567409
	Ovarian cancer	Carboplatin + gemcitabine	I/II	DNA damage assay, HRR mutations		NCT02627443
	Ovarian cancer	Gemcitabine	II			NCT02595892
	mCRPC	Carboplatin ± docetaxel	II			NCT03517969
	Advanced solid tumor	Cisplatin + veliparib	I	DNA damage and apoptotic assay	Gr 3/4, 4–31%; 3 pts had PR	NCT02723864
	HNSCC	Cisplatin + XRT	I	DNA damage assay		NCT02567422
	Brain metastases	Whole brain XRT	I	ATR, CHK1, RAD51, cyclin E, DNA-PK assay		NCT02589522
M4344 (oral)	Advanced solid tumor	Carboplatin, gemcitabine, or cisplatin	I			NCT02278250
AZD6738 (oral)	CLL, PLL or B-cell lymphoma	Alone	I	ATR targeted inhibition biomarker		NCT01955668
	HNSCC	Alone	I	TH1/IFNγ gene and TIL state		NCT03022409
	Refractory CLL	Acalabrutinib	I			NCT03328273
	Advanced solid tumor	Paclitaxel	I			NCT02630199
	Advanced solid tumor	Carboplatin, olaparib, or durvalumab	I/II	*ATM* deficiency	Carbo, Gr 3/4, 27–33%; 3 pts had PR Ola, Gr 3/4, 4–7%; 2 pts *BRCA*-mut had PR Dur, no Gr 3/4; 1 pt had PR, 1 had CR	NCT02264678
	TNBC	Olaparib	II	HRR mutations		NCT03330847
	Advanced tumor	Olaparib	II			NCT02576444
	SCLC	Olaparib	II			NCT03428607
	Ovarian cancer	Olaparib	II			NCT03462342
	NSCLC	Durvalumab	II			NCT03334617
	Advanced solid tumor	XRT	I			NCT02223923
BAY1895344	Solid tumor and lymphoma	Alone	I			NCT03188965

*Abbreviations*: *CLL* chronic lymphocytic leukemia, *DDR* DNA damage response, *Gr* grade, *HNSCC* head and neck squamous cell carcinoma, *HRR* homologous recombination repair, *mCRPC* metastatic castration-resistant prostate cancer, *NSCLC* non–small cell lung cancer, *PFS* progression-free survival, *PLL* prolymphocytic leukemia, *PR* partial response, *SCLC* small cell lung cancer, *SD* stable disease, *TNBC* triple-negative breast cancer, *XRT* X-ray radiotherapy

### ATR inhibitors as monotherapy

M6620 (formerly VX-970 or berzosertib), developed by EMD Serono, is a first-in-class ATR inhibitor that has been tested in human models. It has been shown to enhance tumor DNA replication fork collapse when combined with cisplatin and gemcitabine in vitro [27, 29]. M6620 is well tolerated, and no associated dose-limiting toxicities (DLTs) or grade 3/4 adverse events (AEs) were observed during the subsequent phase 1 study [30]. The recommended phase 2 dose (RP2D) for M6620 monotherapy is 240 mg/m^2^ given once weekly via intravenous infusion [31]. CHK1 phosphorylation, a marker for ATR inhibition, was observed to be reduced by 73 to 90% with M6620 in the pre- and posttreatment paired biopsies [31]. One colorectal cancer patient with ATM gene loss achieved complete response and remained on single-agent M6620 for more than 20 months [30]. This clinical observation is consistent with the preclinical data on the synthetic lethality between ATM and ATR inhibitors [13–16]. M4344 (formerly VX-803), an oral ATR inhibitor, is currently under phase 1 evaluation as a single agent and in combination with cytotoxic chemotherapy for advanced solid tumors (NCT02278250).

AZD6738 is an orally active ATR inhibitor developed by AstraZeneca that has been shown in vitro to inhibit ATR kinase activity at IC_50_ of 1 nM and CHK1 phosphorylation at IC_50_ of 74 nM [13]. AZD6738 was shown to suppress both solid and hematological cell lines with an IC_50_ of less than 1 μM [14, 32, 33]. Synthetic lethality was observed with AZD6783 in gastric cancer, non-small cell lung cancer (NSCLC), and chronic lymphocytic leukemia (CLL) cell lines that were ATM deficient [13, 32]. Furthermore, AZD6783 sensitized TP53-deficient CLL cells to chemotherapy and ibrutinib [32]. Combining AZD6738 with WEE inhibitor AZD1775 led to mitosis catastrophe and inactivated Rad 51-mediated homologous recombination in triple-negative breast cancer cell lines [33]. On the basis of these preclinical data, AZD6378 was tested as a monotherapy in a phase 1 study for 11q-deleted or ATM-deficient relapsed/refractory CLL (NCT01955668). One arm of this completed phase 1 study investigated the safety and tolerability of AZD6378 among patients with relapsed/refractory CLL, polymorphic leukemia, or B cell lymphoma. The ongoing phase 1 Patriot study aims to identify the maximum tolerated dose (MTD) of AZD6783 alone and in combination with palliative radiation in advanced-stage solid tumors (NCT02223923) [34].

BAY1895344 is an ATR inhibitor developed by Bayer that is used to inhibit the proliferation of human cancer cell lines with a median IC_50_ of 78 nM. Most sensitive cell lines are characterized by mutations of the ATM-associated pathway [14]. The synergy between BAY1895334 and radium-223, an FDA-approved alpha radiopharmaceutical, was observed in a bone metastasis xenograft model of castration-resistant prostate cancer [14]. The phase 1 study with BAY1895344 in advanced solid tumors and lymphoma is currently at the dose-expansion phase (NCT03188965).

### ATR inhibitors with DNA-damaging cytotoxic chemotherapy

Potential synergy in cell killing was observed when an ATR inhibitor was combined with DNA-damaging chemotherapy in preclinical models. When adding AZD6738 to cisplatin, enhanced cytotoxicity was noted in NSCLC cell lines and cell line xenografts with intact ATM signaling [13]. Potent synergy in cell killing was noted after combining cisplatin with AZD6738 in ATM-deficient NSCLC xenografts [13]. Potential synergies in cancer cell killing were also reported when AZD6738 was combined with cisplatin or gemcitabine in preclinical models of breast cancer [35] and pancreatic cancer [36]. In 2016, Yap et al. reported phase 1 dose escalation data on investigations of AZD6738/carboplatin, AZD6738/olaparib, and AZD6738/durvalumab combinations in advanced solid tumors [37]. AZD6738 at 40 mg given twice daily on days 1 and 2, along with carboplatin at an area under the curve of 5 given on day 1, was the recommended RP2D. Twenty-seven patients were enrolled in the study. Grade 3 toxicities included thrombocytopenia (36.4%), neutropenia (27.3%), and anemia (33.3%). Three patients (9.1%) with *ATM*-deficient ovarian, cervical, and rectal cancers achieved partial remission [37]. The dosing and safety of the other two combinations are addressed below.

The ATR inhibitor and carboplatin (area under the curve of 5 at day 1) combination was also tested with M6620 in a phase 1 study reported by O’Carrigan et al. [31]. M6620 given intravenously on days 2 and 9 of a 21-day cycle was tolerated at a higher dose (90 mg/m^2^) with fewer associated grade 3/4 AEs than oral AZD6738. Grade 3/4 neutropenia was observed in four patients (21%), and grade 3/4 thrombocytopenia was reported in one (5%). One patient with BRCA1-mutated, platinum-refractory, PARP inhibitor–resistant ovarian cancer achieved partial response (PR) for 6 months [31, 38]. Preliminary data for the dose-expansion cohort of the M6620/cisplatin combination (NCT02157792) were presented at the San Antonio Breast Cancer Symposium in December 2017. In this expansion cohort, patients with metastatic triple-negative breast cancer (TNBC) were given M6620 at 140 mg/m^2^ on days 2 and 9 and cisplatin at 75 mg/m^2^ on day 1 of every 21-day cycle. Thirty-five females were enrolled in this study, 18 of whom were confirmed to have BRCA1/2 wild-type TNBC. Median progression-free survival was 4.1 months, and preliminary unconfirmed objective response was 38.9%. Grade 3/4-related AEs occurred among 16 patients (45.7%) [39].

M6620 was also tested in combination with gemcitabine in a phase 1 study on advanced solid tumors. The RP2D and schedule were M6620 given at 210 mg/m^2^ on days 2, 9, and 16 along with gemcitabine at 1000 mg/m^2^ given on days 1 and 8 of each 21-day cycle. Grade 3/4 toxicity was observed in 25 out of 50 patients. PR was noted in one out of four breast cancer cases and in one out of six NSCLC cases. Median progression-free survival ranged from 8.3 to 29.3 weeks (NCT02157792) [40].

Enhanced sensitivity to a topoisomerase I inhibitor was observed in ATR-depleted preclinical models. On the basis of this observation, Thomas et al. conducted a phase 1 study (NCT02487095) in which M6620 combined with topotecan was used to treat 21 patients with advanced solid tumors [41]. The maximal planned dose was reached with M6620 at 210 mg/m^2^ given on days 2 and 5 plus topotecan at 1.25 mg/m^2^ given on days 1 to 5 of every 21-day cycle. Grade 3/4 toxicities were mainly myelosuppression related, including anemia (19%), leukopenia (19%), neutropenia (19%), lymphopenia (14%), and thrombocytopenia (10%). Two cases of PR and seven cases of stable disease (SD) were observed, which accounted for the 42.8% disease control rate. Moreover, three out of five patients (60%) with platinum-refractory small-cell lung cancer had PR or prolonged SD [41]. Pharmacodynamic studies showed preliminary evidence of enhanced DNA double-strand breaks in response to this combination.

As in the clinical development of the PARP inhibitor/DNA-damaging chemotherapy combination, cytopenias were the main grade 3/4 toxicities in early-phase trials combining an ATR inhibitor with cytotoxic chemotherapy. Nevertheless, M6620 was better tolerated when combined with a topoisomerase inhibitor than with platinum chemotherapy. Small-cell lung cancer is characterized with high frequency of p53 and Rb1 loss, genomic instability, and high mutation burden [42]. The disease is either refractory to platinum-based chemotherapy, or more often, initially responds to the treatment but subsequently becomes resistant. The refractory/resistance mechanism is not fully understood. The mutation status of P53 and Rb1 might be associated with refractory/resistance to chemotherapy [43]. Other factors including SLFN11 gene silencing are also considered to be a potential mechanism of chemoresistance [44]. The combination of M6620 with a topoisomerase inhibitor seems to have great potential in platinum-refractory/resistance small-cell lung cancer.

This use of this combination in platinum-refractory small-cell lung cancer would be a breakthrough in the treatment of this rare and aggressive cancer.

### ATR inhibitors with radiotherapy

Ionizing radiation is known to cause a variety of DNA damages, including double-strand and single-strand DNA breaks. To repair radiation-induced DNA damage, cell cycle checkpoint activation is required, as it facilitates the time necessary for damaged-DNA reparations. Blocking ATR activity would not only impair DNA repair but would also block cell cycle checkpoint activation. This double blockade in the DDR against ionizing radiation has been proven to be effective in cancer cell killing in several preclinical models. AZD6738 was shown to radiosensitize multiple cancer cell lines regardless of the p53 and BRCA status [45]. A similar effect was observed with M6620 in pancreatic cancer [46] and lymphoma [47] cell lines. The phase 1 study PATRIOT (NCT02223923) uses a 3 + 3 design to test dose escalation of AZD6738, first as a monotherapy and then in combination with 20 Gy in ten fractions of palliative radiotherapy. The radiation dose will be escalated after the MTD of AZD6738 is reached. The expansion cohorts will add maintenance doses of AZD6738 until disease progression [34]. M6620 is also being studied in combination with whole brain radiation among patients with brain metastases from NSCLC (NCT02589522) and in combination with concurrent chemoradiation with cisplatin for head and neck squamous cell carcinoma (NCT02567422).

### ATR inhibitors with immune checkpoint inhibitors

Emerging preclinical evidence indicates that ATR inhibitors can block the programmed death-ligand 1 upregulation on cancer cell surfaces and mitigate the tumor infiltration of regulatory T cells after treatment with radiation or cisplatin [48]. The safety and tolerability of combining ATR inhibitor with anti-programmed death-ligand 1 therapy are being tested in an ongoing phase 1 study [37]. In this study, AZD6738 was given at 80 mg twice daily during the 14-day monotherapy run, which was followed by AZD6738 given on days 22 to 28 concomitantly with durvalumab 1500 mg on days 1 and 28. One patient with squamous cell carcinoma of the larynx achieved PR, and one patient with NSCLC potentially obtained complete response [37]. This dose and schedule were well tolerated with no DLTs observed. This combination is being evaluated in a phase 2 multi-arm umbrella study specifically for ATM-deficient NSCLC [49]. On the basis of having a good tolerability, the ATR inhibitor/immune checkpoint inhibitor combination could be developed as a trimodality therapy by adding treatments such as ionizing radiation.

### ATR inhibitors with PARP inhibitors

RNA interference (RNAi)-mediated depletion or inhibition of ATR has been shown to sensitize ovarian cancer cells to cisplatin, topotecan, gemcitabine, and the PARP inhibitor veliparib (ABT-888) [50]. Moreover, an ATR inhibitor further enhanced the killing of BRCA1-depleted ovarian cancer cells by cisplatin, topotecan, and veliparib [50]. Amplification of ATR and CHK1 genes was noted in ovarian cancers with genomic instability. Inactivating Rad51 in the homologous recombination repair (HR) pathway led to differential sensitivity of MCF-7 and Hela cells to ATR and CHK1 inhibitors, implicating ATR and CHK1 as potential drug targets for HR-defective cancers [51]. More recently, ATR was shown to control the abundance of HR factors, largely via CHK1-dependent transcription and promotion of specific HR protein stabilization. Long-term inhibition of ATR signaling severely impaired the ability of cells to use HR-mediated DNA repair [52]. Collectively, these preclinical studies provide the rationale for using an ATR inhibitor/PARP inhibitor combination in HR-proficient and HR-deficient cancer cells.

Twenty-seven patients were enrolled in the AZD6738 and olaparib arm of the phase 1 AZD6738 combinational study reported by Yap et al. at the 2016 EORTC-NCI-AACR Molecular Targets and Cancer Therapeutics Symposium [37]. The RP2D included AZD6738 at 160 mg daily from days 1 to 7 and olaparib at 300 mg twice a day from days 1 to 28. Two patients with BRCA-mutant TNBC achieved PR with this regimen [37]. M6620 was evaluated in combination with veliparib and cisplatin in a phase 1 study, with the intention to induce a BRCA null-like phenotype. When the preliminary data were reported in 2018, the MTD was not yet reached and the study was enrolling at dose level (DL) 7 with cisplatin at 40 mg/m^2^ on days 1 and 8, M6620 at 210 mg/m^2^ on days 2 and 9, and veliparib at 200 mg taken orally twice daily from days 1 to 3 and 8 to 10 [53]. The associated grade 3/4 AEs included hypophosphatemia (4%), thrombocytopenia (31%), leukopenia (22%), and lymphopenia (18%). PR was achieved in 3 out of 22 patients (13.6%), including 1 with BRCA wild-type ovarian cancer. SD was observed in 12 out of 22 patients (54.5%) [53].

Further clinical investigation of the PARP inhibitor/ATR inhibitor combination has been extended to multiple phase 2 trials, including the VIOLETTE study on TNBC [54], the SUKSES-N2 study on small-cell lung cancer (NCT03428607), and the CAPRI study on ovarian cancer (NCT03462342). The VIOLETTE study will be stratified on the basis of HR gene alterations and, the CAPRI study will be stratified on the grounds of platinum sensitivity. The OLAPCO study (NCT02576444) is a biomarker-enriched multi-arm olaparib-based combination study. The AZD6738 and olaparib arm requires the preselection of tumors with mutations in HR-DNA repair genes.

## Conclusion

Although the ATR-CHK1 pathway in DDR has been studied for decades, it was not until recently that the small molecule inhibitors of ATR were developed for the clinical setting [55]. Inhibition of ATR with an ATR inhibitor either as a monotherapy or in combination with DNA- damaging chemotherapy drugs, ionizing radiation, immune checkpoint blockers, or PARP inhibitors is being tested in early-phase clinical trials in advanced solid tumors and hematological malignancies. Safety and tolerability have been reported for M6620 and AZ6738. Phase 2 combination trials are ongoing. Emerging data from these early-phase studies support the preclinical observations of the synthetic lethality of ATR inhibitors in ATM-deficient cancers. Currently, there are no data on whether lack of functional p53 or the replication stress induced by overexpression of oncogenes such as c-MYC can serve as predicative biomarkers for ATR inhibitor monotherapy. Other than predicative biomarkers, data coming from ATR inhibitor-based combinational trials could shed light on whether we can expand this therapy to HR-proficient cancers and whether this approach can serve as a rescue therapy for patients who have progressed through PARP inhibitors.

## Abbreviations

ATM: Ataxia telangiectasia mutated
ATR: Ataxia telangiectasia and Rad3 related
ATRIP: ATR interacting protein
CHK1: Checkpoint kinase 1
CHK2: Checkpoint kinase 2
CLL: Chronic lymphocytic leukemia
DDR: DNA damage response
HR: Homologous recombination repair
MTD: Maximum tolerated dose
NSCLC: Non-small cell lung cancer
PARP: Polymerase
PR: Partial response
RNAi: RNA interference
SD: Stable disease
ssDNA: Single-strand DNA
TNBC: Triple-negative breast cancer
